# Are Changes in Neighbourhood Perceptions Associated with Changes in Self-Rated Mental Health in Adults? A 13-Year Repeat Cross-Sectional Study, UK

**DOI:** 10.3390/ijerph14121473

**Published:** 2017-11-29

**Authors:** Jonathan R. Olsen, Ruth Dundas, Anne Ellaway

**Affiliations:** MRC/CSO Social and Public Health Sciences Unit, University of Glasgow, 200 Renfield Street, Glasgow G2 3QB, UK; ruth.dundas@glasgow.ac.uk (R.D.); anne.ellaway@glasgow.ac.uk (A.E.)

**Keywords:** neighbourhoods, neighbourhood perceptions, depression, anxiety, mental health, environment, health inequality

## Abstract

The aim of this study was to examine changes in neighbourhood perceptions on self-rated mental health problems over time, and to explore demographic, geographic and socio-economic factors as determinants of increased or decreased anxiety and depression symptoms. We conducted a repeat cross-sectional study of individuals (N: 4480) living in the same areas of west central Scotland in 1997 and 2010. Individuals were asked to complete a questionnaire at both time-points, containing 14 questions relating to neighbourhood perceptions and the Hospital Anxiety and Depression Scale (HADS). A three-level linear regression model was fitted to HADS scores and changes in neighbourhood perceptions over time; controlling for a number of individual and area-level variables. Overall, area-level mean HADS scores decreased from 1997 to 2010. When adjusted for individual and area-level variables, this decrease did not remain for HADS anxiety. Applying an overall 14-scale neighbourhood perception measure, worsening neighbourhood perceptions were associated with small increases in depression (0.04, 95% confidence interval (CI) 0.01 to 0.07) and anxiety (0.04, 95% CI 0.00 to 0.08) scores over time. This highlights a need for local and national policy to target areas where neighbourhood characteristics are substantially deteriorating in order to ensure the mental health of individuals does not worsen.

## 1. Introduction

A number of studies have shown that the residential environment is associated with mental health, after taking individual characteristics into account such as age, gender and socio-economic status [1]. Poorer mental health has been associated with a wide range of neighbourhood built-environment factors, such as: traffic hazards, lower street connectivity and land-use diversity [2,3]. Studies have also shown that how people experience their local neighbourhood is associated with their mental health. For example, higher levels of perceived neighbourhood problems such as housing quality, amount of greenspace, land-use mix, industrial activity and traffic volume, have been linked to poorer mental health [4]. However, most studies to date have been cross-sectional and few have examined if changes in neighbourhood perceptions matter for health outcomes such as depression and anxiety, within the same populations, geographical areas and over time.

Globally, the number of individuals suffering from anxiety and/or depression rose by 50% from 1990 to 2013; totalling 615 million individuals in 2013 [5]. If a similar increase in diagnoses continued to 2030, the estimated global healthcare treatment costs would be US$147 billion [5]. However, there are other interventions that could improve mental health, such as improvements to the neighbourhood environment; identifying modifiable factors that could strengthen mental health is crucial to informing the development of interventions to improve mental health [6].

Neighbourhood perceptions can adversely impact upon how individuals interact with the local environment and it is important to consider that neighbourhoods are not characterised by singular static attributes across the social and built environment, as both the social and built environment can change over time. As neighbourhoods change there may be a mismatch between residents’ needs and preferences that can only be resolved by moving to areas which suits their needs [7]. However, households on low income make locational choices within a restricted choice set [8] and individuals can remain resident in areas that can become more or less desirable, influencing residential stress, or in areas that are perceived to remain fundamentally the same. Perceptions of the neighbourhood may change over time as individuals go through their life course. Therefore, it is important to incorporate both individual and area-level determinants when assessing the impact of neighbourhood change [9].

Although perceptions of place can be a significant predictor of individuals’ health, perception of place can differ within a neighbourhood [10]. Studies have shown that between 30% to 38% of the unexplained variance in mental health and well-being scores could be explained by individual factors and perceived community characteristics [11]. The impact of changing neighbourhoods can differ by individual; research in the Netherlands, for example, revealed that changes in the socio-economic status of an area did not impact upon the wish to move, however, increases in the proportion of non-white ethnic minorities within a neighbourhood did increase the number of residents wishing to leave [7]. Neighbourhood-level stresses, such as crime and safety, can lead to additional health problems later in life; remaining after adjustment for individual and area-level socio-economic status [12]. The ‘neighbourhood’ effect can also be influenced by the size and structure of the area under investigation; for example, a smaller administrative boundary may be a more accurate measure of place due to considerable variation in outcomes of individuals living in the same area. It is important to compare the sensitivity of varying neighbourhood definitions to residents’ health systematically [13].

The aim of our study was to examine changes in self-rated neighbourhood perceptions and self-rated mental health problems over time for individuals living in the same geographical areas, and to explore demographic, geographic and socio-economic factors as determinants of increased or decreased anxiety and depression scores over time.

## 2. Materials and Methods

### 2.1. Study Population

The data used were from our 2010 and 1997 postal survey ‘*Transport, Housing and Well-being’* (THAW) of a random stratified sample of adults in eight local authority areas in the west of Scotland. THAW draws on respondents from the same geographical areas (due to its socially heterogeneous composition) and uses a very similar postal questionnaire in both waves [14,15]. Our random sample of the general population was stratified to reduce selection bias [16] using a geodemographic classification of neighbourhood type (using ACORN, Scottish version [17]) to ensure that all types of residential neighbourhoods (ranging from ‘affluent consumers in large houses’ to ‘poorest council estates’) were included in correct proportions.

The 2010 postal questionnaire (see Supplementary Materials), with three reminders (using Dillman’s total design method [18]) achieved a response rate of 38% (2092 completed questionnaires, of whom 68 were excluded from the present analyses due to missing postcode data), from a sample of 5521 adults drawn from the electoral roll in the eight local authority areas which make up the Glasgow and Clyde Valley Structure Plan area in the west of Scotland. The estimated population in this area in 2010 was 1,763,430, and contains marked variations in social status and in health [19]. Survey respondents’ ages ranged from 17 to 95 years old. The socio-demographic characteristics of THAW 2010 were comparable to the previous THAW 1997 Study; e.g., respondents’ own social class was similar in THAW 1997 and THAW 2010 (65% and 70% in the non-manual social class groups, respectively). Compared to the west central Scotland population, our achieved study sample characteristics were broadly similar for sex and for age; 56% were female, and 65% were of working age (18 to 60 years old), compared to 52% and 62% respectively within west central Scotland [20]. Within our sample, 85% of respondents had access to at least one car or van, while within the 2010 Scottish Household Survey, within west central Scotland, 70% had access to a car (does not include van access) [21].

THAW 2010 was approved by the Ethics Committee of the Faculty of Law, Business and Social Sciences at the University of Glasgow.

### 2.2. Outcome Variable—Hospital Anxiety and Depression Scale (HADS)

Individuals were asked to complete the 14-item Hospital Anxiety and Depression Scale (HADS) [22], a common measure of psychological distress that has been in use for over 30 years [23]. The HADS was analysed separately, as two seven-item scores, to provide a measure of both anxiety and depression; a higher score indicated greater reported symptoms. Anxiety and depression are usually scored separately and interpreted using the following matrix: 0–7, none case; 8–10, mild case; 11–14, moderate case; and 15–21, severe case [24].

### 2.3. Independent Variables

A number of individual-level variables were collected; this paper analysed sex, age (in years), self-rated health status over the past 12 months, limiting longstanding illnesses (LLSI), employment status, housing tenure and household car access. Age (in years) was grouped into the following four categories for analysis: 17 to 24, 25 to 44, 45 to 64 and 65+; health status was dichotomised into ‘excellent/good’ or ‘fair/poor’; and housing tenure was dichotomised as ‘owner occupied’, ‘private renter’, or ‘social renter’. Respondents with missing data for independent, dependent or control variables, were recorded as missing and included within each model; 6.5% of individuals having incomplete HADS scores. We tested for missing completely at random (MCAR) using Little’s MCAR test [25]; when auxiliary variables were included Little’s MCAR test results were not significant (*p* = 0.590), the data were therefore assumed to be MCAR. We derived social class from individual responses to own occupation, this was subsequently classified using the registrar general’s six-fold classification [26]. Each participant was linked to the six-category Scottish urban/rural index [27] and the 2012 Scottish Index of Multiple Deprivation (SIMD) quintile (1 = most deprived, 5 = least deprived) [28] based and attached at on the home postcode level. The SIMD combines 38 indicators across 7 domains, such as: income, employment, health, education, skills and training, housing, geographic access and crime.

### 2.4. Neighbourhood Perceptions

Participants completed 14 questions regarding the area around their home and were asked to report whether it was *a serious problem*, *a minor problem* or *not a problem* (Full item descriptions are included in subsequent paragraph). Individual responses were combined to create an overall neighbourhood perception score, using the following scoring matrix: serious problem = 3, minor problem = 1, not a problem = 0; we included a two-point difference between serious and minor problems to accentuate the difference in perceived neighbourhood problems from minor to serious (Cronbach’s alpha = 0.87). Creating overall scores of neighbourhood factors from categorical responses is a commonly used method in neighbourhood perception studies [29,30].

Two models were performed: a one-dimensional model, grouping all items into a general measure of neighbourhood perceptions; secondly, to identify the underlying constructs of the environment, we performed factor analysis based on participant responses to all 14 neighbourhood perceptions questions. This method is based on our previous work conceptualising neighbourhoods [3,31] and is a commonly applied method in conceptualising and constructing factors of the neighbourhood that may influence health and quality of life [32,33,34]. Three domains of neighbourhood perceptions emerged from the 14 items: *crime and disorder* (comprising: ‘vandalism’, ‘litter & rubbish’, ‘assaults or muggings’, ‘burglary’ and ‘discarded needles or syringes’); *neighbourhood reputation* (‘the people round here’, ‘reputation of neighbourhood’, ‘noise’ and ‘disturbance by children or youngsters’); and *physical environmental problems* (‘uneven or dangerous pavements’, ‘nuisance from dogs’, ‘speeding traffic’ and ‘smell’) (one item, public transport, did not cluster but is included in the overall neighbourhood perception variable). Iterated principal-factor analysis with subsequent varimax and promax rotation was performed in STATA/SE 14.2.

### 2.5. Statistical Analysis

A three-level linear regression model was fitted where individuals (n:4480) were nested within postcodes (n:2026), the smallest plotted geographic boundary in the United Kingdom (UK) containing approximately 15 households [35]; and Scottish data zones (n:1332), which are geographical polygon areas based on the home address of each participant (groups of approximately 750 household residents which respect physical boundaries and natural communities, have a regular shape and contain households with similar social characteristics [36]).

The dependent (outcome variable) was HADS score. Models were performed and reported separately for HADS anxiety and HADS depression. The independent covariates were age, sex, employment status, health status, LLSI, social class, housing tenure, access to a car and neighbourhood perceptions; and urban/rural classification at the postcode/datazone level.

We explored alternative administrative boundaries to examine differences in the resulting variance between levels (i.e., geographical boundaries and the individual) and performed each model systematically using each area-level definition. We found no substantial changes in the variance between the levels, or in the model outputs, as the models were performed. We chose to present a three-level model where individuals were nested within postcodes, then data zones.

All models specified individuals nested within postcodes and data zones as a random effect. Models were fitted subsequently to test the effect of wave, neighbourhood perception, individual and contextual factors, and how they explained the outcome variable. Statistical analyses were completed using MLwinN v2.36 through STATA/SE 14.2 using the *runmlwin* command. Participant characteristics are presented in Table 1, the following models were performed and are presented in Table 2(a,b):

- Model 1: Wave only.
- Model 2: Wave and neighbourhood perceptions; crime and disorder, neighbourhood reputation and physical environmental problems.
- Model 3: Identical to model 2 plus individual-level variables sex, age, employment status, health status, LLSI and social class included.
- Model 4: Identical to model 3 plus housing tenure and contextual variables, urban/rural classification included.
- Model 5: Identical to model 4 plus access to car at household included.
- Model 6 (presented in Table 3): Identical structure to model 5 plus interactions by wave and neighbourhood perceptions.

We examined the unexplained variances at each level following further adjustment in models 1 to 6.

### 2.6. Change in Neighbourhood Perceptions and HADS over Time

Following modelling (model number 5) we computed the linear prediction of HADS anxiety and depression scores for each individual. These were subsequently aggregated to data zones to examine the association between mean change in neighbourhood perceptions (score for all 14 neighbourhood questions) and mean change in depression and anxiety scores overall, by socio-economic status of the data zone (using the Scottish Index of Multiple Deprivation (SIMD) split into quintiles [28]). We report the correlation coefficients to describe the strength and direction of a linear relationship between the two variables, both overall and for each deprivation quintile.

## 3. Results

### 3.1. Participant Characteristics

Participant characteristics by wave are described within Table 1. THAW 1 comprised a total of 2388 individuals who were included in the analysis (41% male), and a smaller sample of 2092 for THAW 2 (44% male). Approximately a quarter of individuals were aged 65-plus in both waves; there was a greater proportion in employment in the later wave (62% 2010, 58% 1997); as were those reporting access to a car (82% 2010, 64% 1997); and excellent/good health (69% 2010, 59% 1997).

### 3.2. Depression

There were no statistically significant differences between HADS depression sex, urbanicity or housing tenure. The younger 17 to 24 age group had substantially higher HADS depression scores than older age groups when adjusted for individual variables. When adjusted for urbanicity, housing tenure and car access there were no differences with those aged over 65 (***models 4 & 5***) (Table 2(a)). In the fully adjusted model (***model 5***), HADS depression scores were higher for those unemployed (1.22 95% confidence interval (CI) 0.73 to 1.71) compared to those employed, and lower for those without access to a car (−0.28 95% CI −2.00 to −0.61) compared to those with.

Worse HADS depression scores were related to health; those who reported fair/poor health (2.31 95% CI 2.05 to 2.57) had higher HADS depression scores compared to those with excellent/good health, and those who reported having no LLSI had lower depression score compared to those with an LLSI (−0.54 95% CI −0.78 to −0.29).

Those who reported worse social environments (0.14 95% CI 0.08 to 0.21) and physical environmental problems (0.10 95% CI 0.04 to 0.15) had higher HADS depression scores; this was not significant for crime and disorder.

### 3.3. Anxiety

There were no statistically significant differences between social class, urbanicity, housing tenure or car access. Females had higher HADS anxiety scores than males (1.19 95% CI 0.94 to 1.44) (Table 2(b)). Older age groups had lower HADS anxiety scores than the younger 17 to 24 age groups; decreasing with increasing age. Worse health was related to higher HADS anxiety scores.

All perceived worsening neighbourhood factors were associated with higher HADS anxiety scores; crime and disorder (0.07 95% CI 0.00 to 0.13), social environment (0.11 95% CI 0.03 to 0.19) and physical environmental problems (0.13 95% CI 0.07 to 0.20).

### 3.4. Overall Change in Neighbourhood Perceptions, and Anxiety and Depression Scores, by Socio-Economic Status

Overall, mean HADS scores for both depression and anxiety reduced from 1997 to 2010; HADS depression reduced from 4.9 to 4.2 and HADS anxiety from 7.1 to 6.6 (Table 1).

The multilevel models (Table 2(a)) showed a decrease in HADS depression scores over time. The reduction halved from −0.62 (95% CI −0.84 to −0.39) to −0.24 (−0.46 to −0.02) when adjusted for individual and contextual variables, and car ownership; but the relationship remained. For HADS anxiety (Table 2(b)), after adjustment for car ownership, individual and contextual variables (models 3–5), the reduction in HADS anxiety over time was no longer significant (model 1: −0.48 (95% CI −0.73 to −0.24), model 5: −0.02 (95% CI −0.28 to 0.24).

There were no changes in HADS anxiety scores by neighbourhood perception over time by individual factors (Table 3). For HADS depression, a small decrease was found where neighbourhood perceptions decreased for two factor groupings: social environment and physical environmental problems. When combining all 14 questions regarding neighbourhood perceptions into a single continuous variable, there was a significant interaction between perceptions and wave (time), underlining a small increase in HADS depression (0.04 95% CI 0.01 to 0.07) and anxiety (0.04 95% CI 0.00 to 0.08) scores as neighbourhood perceptions worsened over time (Table 3). The coefficients represent how a full unit change in the neighbourhood perceptions scale is associated with a change in anxiety or depression.

For both HADS anxiety and depression, most of the unexplained variance was at the individual level. There was little variation in variance estimates between the models for HADS anxiety once adjusted for neighbourhood perceptions (model 2—98% unexplained variance at the individual level). For HADS depression in model 1 (adjusted only for wave), 5.7% of unexplained variance was at the postcode level; when adjusted for neighbourhood perceptions this reduced to 3.8%. However, when adjusted for individual characteristics, 0.5% of unexplained variance was at the postcode level.

**Figure 1:**
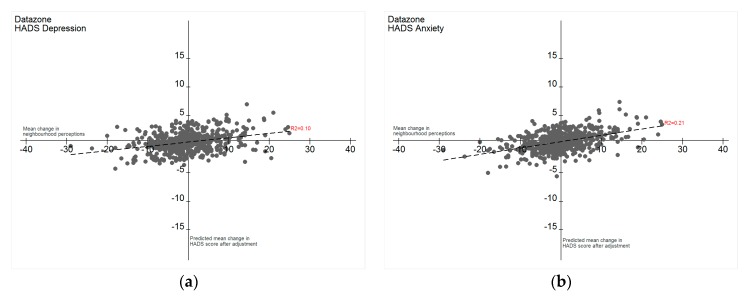
(**a**) Change in overall neighbourhood perceptions and predicted change in HADS depression, over time; (**b**) change in overall neighbourhood perceptions and predicted change in HADS anxiety, over time.

Figure 1a,b highlight a small positive linear relationship between change in neighbourhood perceptions and predicted change in HADS scores (depression: r^2^ = 0.10, anxiety: r^2^ = 0.21) over time, by data zone. The relationship is stronger for HADS anxiety than depression, in the most deprived areas, decreasing as areas became less deprived, except for those living in quintile 4 (second least deprived) which showed the strongest relationship (Table 4).

## 4. Discussion

The aim of our study was to examine changes in neighbourhood perceptions, HADS depression and anxiety scores among residents living in the same areas of Scotland over a 13-year time period (1997 to 2010). Overall, mean HADS depression and anxiety scores decreased over this period; however, when adjusted for individual and area-level variables, including housing tenure and car access, this change was significant for depression only. Increased HADS depression scores were associated with older age, unemployment, fair/poor health, having an LLSI, and no car access. Increase HADS anxiety scores were associated with female sex, younger age, unemployment, fair/poor health, and having a LLSI. 

Worsening neighbourhood perceptions, when grouped by three main factors (crime and disorder, social environment and physical environmental problems), did not result in worsening HADS anxiety scores over time. However, for depression, worse social environment and physical environmental problems did show small decreases in scores. Systematic reviews of neighbourhood characteristics and depression have shown that social processes show the greatest relationship with depression [37]. For anxiety this is more complex, studies have shown associations in both directions —that compositional or contextual explanations are related to areas factors and anxiety [38], highlighting that for anxiety symptoms and neighbourhood problems the relationship requires further understanding. When using an overall neighbourhood perception measure, worsening neighbourhood perceptions were associated with increased depression and anxiety scores. This suggests that a combination of multiple worsening neighbourhood issues increase self-rated depression and anxiety scores over time; this remained after adjustment for individual and area-level characteristics. For HADS depression, worsening neighbourhood perceptions around physical environmental problems and the social environment were associated with higher scores. For HADS anxiety, this also included problems related to crime and disorder. Although our results highlight that a unit change in worsening neighbourhood perceptions are significantly associated with increased anxiety and depression scores, the change represented only a small, yet significant increase in both HADS anxiety and depression symptoms (0.04). When these increases are interpreted using the HADS scoring matrix [24], they are unlikely to lead to what may be classified as *clinically important* worsening anxiety or depression, unless there is substantial worsening of all neighbourhood factors included in the 14-item scale. However, for individuals living in areas where there are worsening neighbourhood perceptions, they are unlikely to experience improved anxiety and depression scores that are experienced overall at a population level, potentially widening health inequalities.

Studies measuring change in the built environment and the impact on health are limited. This may be due to difficulties in effectively measuring neighbourhood change and subsequent impact on health. Recently, there have been studies exploring the feasibility of using historical data to describe objective changes in places over the life course and the relationship with health outcomes [39]; highlighting that this is, indeed, feasible but technically challenging. Alternatively, subjective measures of change in the built environment provide data of individual perceptions of changes to the social and urban landscape. Although, these may be subjective and over shorter time periods, worsening neighbourhood aspects could have impacts on mental health over small time periods, such as months or years. When comparing neighbourhood perceptions to objective measures, these may not always be correlated, but perceptions of neighbourhood disorder can have a greater impact on mental illness than objective measures [40]. Our study examined neighbourhood perceptions at two time points over a 13-year time period. Other studies have examined various time periods and have shown change in perceptions of neighbourhoods; for example, a US study examining the impact of moving to low- or high-income areas on health over a three-year time period [41] and found individuals who moved to low-poverty neighbourhoods reported less distress than those who remained in high-poverty neighbourhoods. A study in Midwest, USA, showed that the development of one or two new health conditions for individuals living in the same area over a 10-year period were lower for every $10,000 increase in neighbourhood income, and there was no variation by age or housing tenure [42]. In a UK study, individuals were followed up over a seven-year period in south Wales; living in more socially cohesive neighbourhoods was strongly associated with improvement in mental health scores, compared to those in low social-cohesive neighbourhoods [43]. A four-year longitudinal study of children in Germany found that the neighbourhood environment (street type, traffic volume and walkability) was associated with increases in childhood BMI over time. However, family and social factors had a greater impact on change in BMI than the neighbourhood built and social environment [44]. We found no studies that examined the impact of shifting cultural norms upon neighbourhood perceptions over time.

We found the strongest relationship between worsening neighbourhood perceptions and increased HADS depression and anxiety scores in quintile 4, the second least deprived quintile. Another study that examined the distribution of services and greenspace in Glasgow found that same deprivation quintile 4, which contained many non-residential dwellings, had the second least access to greenspace, after the most deprived areas, and tended to be closer to business districts and contained most services (i.e., dental and ophthalmic practices, banks, building societies, pawnbrokers, ATMs, cafes, museums/art galleries, railway and subway stations, private health clubs) [45]. Our findings that worsening mental health for those living in quintile 4 (second most deprived) may be due to changes in the built environment over the study time frame and we will investigate this further.

A key strength of our study was that we asked individuals from the same geographical areas to complete the same questions about neighbourhood perceptions and health 13 years apart. A further strength of our study is that we were able to include individual level socio-economic status variables, which has been an important methodological flaw of many other studies that apply census-level attributes [46].

The cross-sectional study design does have limitations and it is important that caution is applied when assessing change over time for outcomes measured by not necessarily the same individuals at each time point [47], as we did in our study. To ensure we did not simply capture population-level trends over time, we used a modelling strategy that added individual and area-level contextual variables to adjust for these factors using a systematic method. The cross-sectional design only allows us to describe associations and not infer causality between neighbourhood perceptions, anxiety and depression. Longitudinal cohort studies using life-course approaches, such as a recent study by Cherrie et al. (2018), may provide more evidence for exposures over the life course and mental health outcomes [48].

Our analysis used a three-level linear regression model; this allowed us to complete the analysis accounting for variance at an area level. An appropriate area-level variable was chosen based on the number of individuals nested within. Studies have highlighted that it is important to consider the size and structure of the neighbourhood when exploring variance between levels and when defining a neighbourhood. Our dataset included individuals at the lowest aggregation (full UK postcode, containing ~15 households) which allowed us to aggregate upwards systematically evaluating several different administrative boundaries (from approximately 15 to 4000 individuals) as levels in our modelling strategy. However, as with other studies [13,49], our findings showed relatively no difference in unexplained variance between these area levels. As Mitchell (2001) highlighted in his critique of multilevel models in determining neighbourhood effect, it is important not to entirely distinguish between neighbourhood or individual effects based solely on the variance between multi-level models [50]. However, we found possible differences between the anxiety and depression scales at the smaller (postcode) area level, suggesting a more immediate neighbourhood effect on depression, but this difference disappeared in the fully adjusted model 6. These relations are complex, where both the neighbourhood impacts on individuals and individuals impact on neighbourhoods [51]. We recommend future research explores the relationship between individual factors (such as age and employment), neighbourhood perceptions, anxiety and depression.

## 5. Conclusions

Our 13-year repeat cross-section study of adults in the west of Scotland found that worsening perceived neighbourhood factors were associated with increased symptoms of anxiety and depression. Our findings highlight that local and national policy makers must target areas where there may be substantially deteriorating neighbourhood perceptions, particularly where physical environmental problems, social environments, and crime and disorder are perceived to be worsening, in order to ensure that individuals do not develop poorer mental health.

## Figures and Tables

**Table 1:** Participant characteristics and Hospital Anxiety and Depression Scale (HADS) score.

Variable	THAW I (N = 2388)		THAW II (N = 2092)	
	N	%	N	%
**Sex**				
Male	985	41.39	911	43.55
Female	1395	58.61	1181	56.45
**Age**				
17 to 24	95	3.98	112	5.35
25 to 44	859	35.97	504	24.09
45 to 64	788	33.00	943	45.08
65-plus	646	27.05	533	25.48
**Urban/Rural Classification**				
Large Urban Areas	1567	65.62	1204	59.49
Other Urban Areas	594	24.87	539	26.63
Accessible Small Towns	147	6.16	175	8.65
Accessible Rural	74	3.10	92	4.55
Remote Rural	6	0.25	14	0.69
**Social Class**				
I/II Professional, managerial and technical	601	32.70	820	44.10
III Skilled	531	28.90	737	39.60
IV/V Partly skilled and unskilled	705	38.40	302	16.30
**Employment**				
Employed or student	1139	57.50	1186	62.13
Unemployed	129	6.50	100	5.24
Retired	712	36.00	623	32.63
**Housing Tenure**				
Owner occupied	1478	64.32	1711	83.22
Private Renter	47	2.05	60	2.92
Social Renter	773	33.64	285	13.86
**Car Access at Household**				
Access	1452	64.10	1697	81.50
No Access	813	35.90	384	18.50
**Health Status**				
Excellent/Good	1410	59.05	1444	69.02
Fair/Poor	978	40.95	648	30.98
**LLSI**				
Has LLSI	1133	47.45	956	45.70
No LLSI	1255	52.55	1136	54.30
**HADS Depression**				
	Mean (sd ^1^)	Median (IQR ^2^)	Mean (sd ^1^)	Median (IQR ^2^)
	4.9 (3.8)	4 (2–7)	4.2 (3.8)	3 (1–6)
**HADS Anxiety**				
	Mean (sd ^1^)	Median (IQR ^2^)	Mean (sd ^1^)	Median (IQR ^2^)
	7.1 (4.0)	7 (4–9)	6.6 (4.1)	6 (3–9)
**Neighbourhood Perceptions Scale**				
	Mean (sd ^1^)	Median (IQR ^2^)	Mean (sd ^1^)	Median (IQR ^2^)
	9.5 (7.5)	8 (4–13)	8.0 (7.0)	6 (3–11)

^1^ sd: standard deviation; ^2^ IQR: interquartile range; Percentages may not total 100% due to rounding.

**Table 2:** (**a**) Multilevel linear regression analyses of HADS depression, wave, individual level variables, contextual level variables and neighbourhood perceptions. (**b**) Multilevel linear regression analyses of HADS anxiety, wave, individual level variables, contextual level variables and neighbourhood perceptions.

**(a)**																				
**HADS Depression**	**Model 1: Wave Only**				**Model 2: Wave and Neighbourhood Perceptions**				**Model 3: Model 2 + Individual Variables**				**Model 4: Model 3 + Contextual Variables**				**Model 5: Model 4 + Car Access**			
	**Coef**	***p***	**LL 95% CI**	**UL 95% CI**	**Coef**	***p***	**LL 95% CI**	**UL 95% CI**	**Coef**	***p***	**LL 95% CI**	**UL 95% CI**	**Coef**	***p***	**LL 95% CI**	**UL 95% CI**	**Coef**	***p***	**LL 95% CI**	**UL 95% CI**
**Sex**																				
Male									REF				REF				REF			
Female									0.11	0.31	−0.10	0.32	0.13	0.23	−0.08	0.34	0.12	0.28	−0.09	0.33
**Age**																				
17 to 24									REF				REF				REF			
25 to 44									0.56	0.02	0.08	1.04	0.57	0.02	0.09	1.06	0.59	0.02	0.10	1.07
45 to 64									0.66	0.01	0.18	1.15	0.66	0.01	0.17	1.15	0.67	0.01	0.18	1.16
65-plus									0.64	0.04	0.02	1.27	0.68	0.04	0.05	1.31	0.64	0.05	0.00	1.27
**Employment**																				
Employed or Student									REF				REF				REF			
Unemployed									1.32	<0.001	0.85	1.79	1.22	0.00	0.74	1.70	1.22	<0.001	0.73	1.71
Retired									−0.05	0.79	−0.45	0.35	−0.11	0.59	−0.52	0.29	−0.13	0.52	−0.54	0.27
**Health status**																				
Excellent/Good									REF				REF				REF			
Fair/Poor									2.43	<0.001	2.17	2.68	2.35	0.00	2.08	2.61	2.31	<0.001	2.05	2.57
**LLSI**																				
Has LLSI									REF				REF				REF			
No LLSI									−0.51	<0.001	−0.74	−0.27	−0.53	0.00	−0.77	−0.29	−0.54	<0.001	−0.78	−0.29
**Social class**																				
I/II Professional, Managerial and Technical									REF				REF				REF			
III Skilled									0.36	<0.001	0.12	0.60	0.38	<0.001	0.14	0.62	0.37	<0.001	0.13	0.62
IV/V Partly Skilled and Unskilled									0.16	0.24	−0.11	0.44	0.15	0.30	−0.13	0.43	0.12	0.41	−0.17	0.41
**Urban/rural classification**																				
Large Urban Areas													REF				REF			
Other Urban Areas													0.06	0.64	−0.19	0.31	0.05	0.71	−0.21	0.30
Accessible Small Towns													−0.03	0.87	−0.45	0.38	0.00	1.00	−0.42	0.42
Accessible Rural													0.27	0.38	−0.33	0.88	0.31	0.32	−0.30	0.92
Remote Rural													−0.01	0.99	−1.57	1.56	0.02	0.98	−1.55	1.59
**Housing Tenure**																				
Owner Occupied													REF				REF			
Private Renter													−1.20	<0.001	−1.89	−0.52	−1.31	<0.001	−2.00	−0.61
Social Renter													0.33	0.05	0.01	0.66	0.24	0.17	−0.10	0.58
**Car access at household**																				
Access																	REF			
No Access																	−0.28	0.07	−0.58	0.02
**Neighbourhood perceptions**																				
Crime and Disorder					0.03	0.32	−0.03	0.08	−0.03	0.29	−0.08	0.02	−0.03	0.33	−0.08	0.03	−0.03	0.34	−0.08	0.03
Social Environment					0.27	<0.001	0.21	0.34	0.15	<0.001	0.08	0.21	0.14	<0.001	0.08	0.21	0.14	<0.001	0.08	0.21
Physical Environmental Problems					0.13	<0.001	0.08	0.19	0.11	<0.001	0.06	0.16	0.10	<0.001	0.05	0.15	0.10	<0.001	0.04	0.15
**THAW wave**																				
Wave 1	REF				REF				REF				REF				REF			
Wave 2	−0.62	<0.001	−0.84	−0.39	−0.50	<0.001	−0.72	−0.28	−0.27	0.02	−0.48	−0.05	−0.25	0.03	−0.47	−0.03	−0.24	0.04	−0.46	−0.02
Cons	4.87	<0.001	4.70	5.03	3.80	<0.001	3.58	4.02	2.37	0.00	1.80	2.94	2.37	<0.001	1.79	2.95	2.65	<0.001	2.00	3.30
**Variance**	**ICC**	**S.Err.**	**LL 95% CI**	**UL 95% CI**	**ICC**	**S.Err.**	**LL 95% CI**	**UL 95% CI**	**ICC**	**S.Err.**	**LL 95% CI**	**UL 95% CI**	**ICC**	**S.Err.**	**LL 95% CI**	**UL 95% CI**	**ICC**	**S.Err.**	**LL 95% CI**	**UL 95% CI**
Level 3: datazone	5.1%	0.29	0.16	1.29	3.0%	0.25	−0.10	0.89	3.5%	0.19	−0.08	0.68	3.6%	0.19	−0.08	0.68	3.8%	0.19	−0.06	0.70
Level 2: postcode	5.7%	0.37	0.09	1.53	3.8%	0.33	−0.15	1.15	0.5%	0.25	−0.44	0.53	0.0%	0.25	−0.49	0.49	0.0%	0.25	−0.49	0.49
Level 1: ID	89.3%	0.36	12.04	13.44	93.3%	0.34	11.73	13.08	96.0%	0.26	7.65	8.68	96.4%	0.27	7.60	8.66	96.2%	0.27	7.58	8.63
**(b)**																				
**HADS depression**	**Model 1: Wave only**				**Model 2: Wave and neighbourhood perceptions**				**Model 3: Model 2 + Individual variables**				**Model 4: Model 3 + contextual variables**				**Model 5: Model 4 + car access**			
	**Coef**	***p***	**LL 95% CI**	**UL 95% CI**	**Coef**	***p***	**LL 95% CI**	**UL 95% CI**	**Coef**	***p***	**LL 95% CI**	**UL 95% CI**	**Coef**	***p***	**LL 95% CI**	**UL 95% CI**	**Coef**	***p***	**LL 95% CI**	**UL 95% CI**
**Sex**																				
Male									REF				REF				REF			
Female									1.17	0.00	0.92	1.42	1.19	0.00	0.94	1.45	1.19	0.00	0.94	1.44
**Age**																				
17 to 24									REF				REF				REF			
25 to 44									−0.11	0.69	−0.68	0.45	−0.15	0.62	−0.72	0.43	−0.13	0.66	−0.70	0.45
45 to 64									−0.79	0.01	−1.36	−0.22	−0.82	0.01	−1.40	−0.24	−0.81	0.01	−1.39	−0.23
65-plus									−1.94	0.00	−2.69	−1.20	−1.89	0.00	−2.64	−1.14	−1.88	0.00	−2.63	−1.12
**Employment**																				
Employed or Student									REF				REF				REF			
Unemployed									0.68	0.02	0.12	1.24	0.68	0.02	0.10	1.26	0.69	0.02	0.10	1.28
Retired									−0.15	0.54	−0.62	0.32	−0.22	0.36	−0.70	0.26	−0.25	0.30	−0.74	0.23
**Health status**																				
Excellent/Good									REF				REF				REF			
Fair/Poor									1.78	0.00	1.47	2.09	1.75	0.00	1.43	2.06	1.72	0.00	1.40	2.03
**LLSI**																				
Has LLSI									REF				REF				REF			
No LLSI									−0.32	0.03	−0.60	−0.03	−0.33	0.03	−0.62	−0.04	−0.34	0.02	−0.63	−0.05
**Social class**																				
I/II Professional, Managerial and Technical									REF				REF				REF			
III Skilled									0.05	0.73	−0.23	0.33	0.07	0.66	−0.22	0.35	0.07	0.64	−0.22	0.36
IV/V Partly Skilled and Unskilled									0.18	0.29	−0.15	0.50	0.15	0.37	−0.18	0.49	0.14	0.43	−0.20	0.48
**Urban/rural classification**																				
Large Urban Areas													REF				REF			
Other Urban Areas													0.21	0.17	−0.09	0.51	0.20	0.19	−0.10	0.50
Accessible Small Towns													−0.10	0.68	−0.59	0.39	−0.09	0.72	−0.58	0.40
Accessible Rural													0.21	0.57	−0.51	0.92	0.23	0.54	−0.49	0.94
Remote Rural													−0.76	0.40	−2.54	1.01	−0.74	0.41	−2.52	1.03
**Housing Tenure**																				
Owner Occupied													REF				REF			
Private Renter													−0.93	0.03	−1.75	−0.12	−1.00	0.02	−1.83	−0.18
Social Renter													0.05	0.82	−0.34	0.43	0.01	0.97	−0.39	0.41
**Car access at household**																				
Access																	REF			
No Access																	−0.18	0.34	−0.53	0.18
**Neighbourhood perceptions**																				
Crime and Disorder					0.10	0.00	0.04	0.16	0.07	0.04	0.00	0.13	0.07	0.04	0.00	0.13	0.07	0.04	0.00	0.13
Social Environment					0.26	0.00	0.19	0.33	0.11	0.01	0.03	0.18	0.10	0.01	0.02	0.18	0.11	0.01	0.03	0.19
Physical Environmental Problems					0.14	0.00	0.08	0.19	0.14	0.00	0.08	0.21	0.14	0.00	0.08	0.21	0.13	0.00	0.07	0.20
**THAW wave**																				
Wave 1	REF				REF				REF				REF				REF			
Wave 2	−0.48	0.00	−0.73	−0.24	−0.30	0.01	−0.53	−0.06	−0.01	0.93	−0.27	0.24	−0.02	0.86	−0.29	0.24	−0.02	0.87	−0.28	0.24
Cons	7.08	0.00	6.91	7.25	5.75	0.00	5.51	5.98	5.41	0.00	4.73	6.08	5.42	0.00	4.73	6.11	5.59	0.00	4.81	6.37
**Variance**	**ICC**	**Std. Err.**	**LL 95% CI**	**UL 95% CI**	**ICC**	**Std. Err.**	**LL 95% CI**	**UL 95% CI**	**ICC**	**Std. Err.**	**LL 95% CI**	**UL 95% CI**	**ICC**	**Std. Err.**	**LL 95% CI**	**UL 95% CI**	**ICC**	**Std. Err.**	**LL 95% CI**	**UL 95% CI**
Level 3: datazone	2.7%	0.30	−0.14	1.03	1.6%	0.26	−0.27	0.75	2.5%	0.18	−0.06	0.65	2.3%	0.19	−0.90	0.64	2.3%	0.28	−0.26	0.83
Level 2: postcode	1.8%	0.39	−0.48	1.05	0.4%	0.34	−0.61	0.74	0.0%	0.00	0.00	0.00	0.0%	0.00	0.00	0.00	0.0%	0.00	0.00	0.00
Level 1: ID	95.5%	0.42	14.68	16.34	98.0%	0.39	13.90	15.43	97.5%	0.34	11.01	12.33	97.7%	0.34	11.01	12.36	97.2%	0.39	10.86	12.37

**Table 3:** Linear change in HADS anxiety and depression scores, neighbourhood perceptions overall and by individual factor groups, over time (1997 to 2010, model 6).

Interactions over Time (1997 to 2010)	HADS Depression				HADS Anxiety			
	Coef	*p*	LL 95% CI	UL 95% CI	Coef	*p*	LL 95% CI	UL 95% CI
**Individual factor groupings by wave**								
Crime and Disorder	0.07	0.06	-0.00	0.14	0.08	0.06	-0.01	0.17
Social Environment	0.13	0.01	0.03	0.22	0.10	0.07	-0.01	0.21
Physical Environmental Problems	0.08	0.05	-0.00	0.16	0.09	0.07	-0.01	0.19
**Overall neighbourhood perception by wave**								
	0.04	0.01	0.01	0.07	0.04	0.04	0.00	0.08

**Table 4:** Correlation coefficients (r^2^) of association between mean change in neighbourhood perceptions and predicted mean change in HADS scores after adjustment by socio-economic status ^1^.

Socio-Economic Status (SIMD Quintile)	Depression	Anxiety
SIMD 1 (most deprived)	0.132	0.292
SIMD 2	0.114	0.247
SIMD 3	0.073	0.175
SIMD 4	0.183	0.348
SIMD 5 (least deprived)	0.005	0.022

^1^ Analysis aggregated to data zone level to assign area level socio-economic status. Overall measure of neighbourhood perception used in the analysis.

## Supplementary Materials

The following are available online at www.mdpi.com/1660-4601/14/12/1473/s1, File S1: THAW questionnaire.

## Data Sharing Statement

For further information, please refer to the MRC/CSO Social and Public Health Sciences Unit data sharing statement at http://thaw.sphsu.mrc.ac.uk/information-for-researchers.
