# Supplementary material for: Are Changes in Neighbourhood Perceptions Associated with Changes in Self-Rated Mental Health in Adults? A 13-Year Repeat Cross-Sectional Study, UK
Source: Int J Environ Res Public Health. 2017 Nov 29;14(12):1473. doi: 10.3390/ijerph14121473 (PMC5750892; doi:10.3390/ijerph14121473)
Supplement: Supplementary file 1 [file ijerph-14-01473-s001.pdf]

UNIVERSITY of GLASGOW

Medical Research Council MRC

CHIEF SCIENTIST OFFICE

# Transport, Housing and Wellbeing

## *Questionnaire*

*This questionnaire is STRICTLY CONFIDENTIAL and will only be seen by staff working on this project.*

**BARCODE**



INVESTOR IN PEOPLE

MRC/CSO Social and Public Health Sciences Unit, 4 Lilybank Gardens, Glasgow, G12 8RZ
Tel: 0141 357 3949 Fax: 0141 337 2389 www.sphsu.mrc.ac.uk

A Research Unit supported by the Medical Research Council and the Chief Scientist Office of the Scottish Government Health Directorates, at the University of Glasgow

**This questionnaire has four sorts of question.**

**A. The first asks you to indicate the answer that applies to you by ticking a box next to the answer**

For example

Is your home built of sandstone? Yes ☑ No ☐

In the example someone has ticked the box next to "yes" showing that their home is built of sandstone.

**B. The second sort of question asks you simply to write an answer in the boxes provided.**

For example

How many times have you been shopping in the last month? [0] [7]

In the example someone has said that they went shopping 7 times in the last month. If they had not been shopping they would have put 0 in the box.

**C. The third sort of question asks you to circle an answer**

For example

How often do you watch TV in the evenings?

| | | | | |
|---|---|---|---|---|
| ***I watch TV in the evenings*** | *most of the time* | *(a lot of the time)* | *only occasionally* | *never* |

In the example someone has said that they watch TV in the evenings "a lot of the time".

**D. The other sort of question asks you to tell us what you think**

For example

What do you like about holidays?

having a rest

doing something different

In the example someone has said that they like holidays because they can have a rest and they can do something different.

There will be examples to help you answer the questions throughout the questionnaire. Please look out for ☞ to tell you where to go next.

*Don't worry if you tick, write in or circle the wrong answer, just put a line through the incorrect answer and tick, write in, or circle the correct one.*

# About you

**Q1. Over the last 12 months would you say your health on the whole has been excellent, good, fair or poor?**

*Please tick ONE box.* excellent ☐1 good ☐2 fair ☐3 poor ☐4

**Q2. Are you registered as a disabled person?**

*Please tick ONE box.* yes ☐1 no ☐2

**Q3. Over the last 12 months, how many times have you consulted a GP or family doctor on your own behalf?**

This could be you visiting the surgery or the doctor visiting you at home.

*Please WRITE the number of times in the boxes below.*

Number of visits to GP or family doctor ☐☐ time(s) in the last 12 months

**Q4. Are you…?**

*Please tick ONE box.* male ☐1 female ☐2

**Q5. What is your age?**

This information is very important because people of different ages have different needs for housing and transport and also have different health problems.

*Please WRITE your age in the boxes.* ☐☐ years

**Q6. Can we just check, do you still stay at the address this questionnaire was sent to?**

*Please tick 'yes' or 'no' and if you do NOT stay at the same address please write in your new postcode as in the example.*

yes ☐1 no ☐2

| e.g. | | G | 1 | 2 | - | 8 | R | Z |
|---|---|---|---|---|---|---|---|---|
| | | | | | - | | | |

**Q7. Do you have a driving licence?**

*Please tick ONE box.* yes – full ☐1 yes – provisional ☐2 no ☐3

***BARCODE 1*** ***CONFIDENTIAL***

**Q8. On the whole how happy are you with your life in general? Look at the faces and TICK the box under the face which shows best how you feel.**

*Please tick ONE box.*

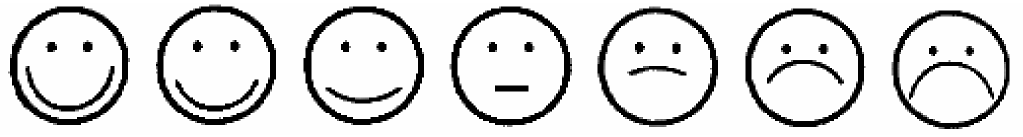

| ☐ 1 | ☐ 2 | ☐ 3 | ☐ 4 | ☐ 5 | ☐ 6 | ☐ 7 |
|---|---|---|---|---|---|---|

## Your health and wellbeing

**Q9. a) Do you have any long-standing illness, disability or infirmity?**

By long-standing we mean anything that has troubled you over a period of time or that is likely to affect you over a period of time.

| *Please tick ONE box.* | yes ☐ 1 | no ☐ 2 |
|---|---|---|

☞ If **NO** go to **Q10** below

☞ If **YES** go to part **b** below

**b) What is the matter with you? Please WRITE in all conditions you have**.

______________________________

______________________________

______________________________

**c) Do any of these illnesses or disabilities limit your activities in any way?**

| *Please tick ONE box.* | yes ☐ 1 | no ☐ 2 |
|---|---|---|

**Q10. Loneliness can be a serious problem for some people and not others. At the present moment do you ever feel lonely?**

*Please tick ONE box.*

| | most of the time | quite often | only occasionally | seldom | never |
|---|---|---|---|---|---|
| **I feel lonely** | ☐ 1 | ☐ 2 | ☐ 3 | ☐ 4 | ☐ 5 |

**Q11. Here is a set of questions about the way you have been feeling in general over the <u>last 7 days</u>.**

The choice of answers is often different for each question, so please read each one carefully and circle the answer which shows how you have been feeling.

For example…

| | | | | |
|---|---|---|---|---|
| ***I feel tired and flat*** | *most of the time* | *a lot of the time* | *only occasionally* | *never* |

The person answering has been feeling tired and flat a lot of the time over the last week, so he or she has circled 'a lot of the time.'

***NOW ANSWER THE QUESTIONS BELOW. PLEASE DON'T MISS ANY OUT.***

| | | | | |
|---|---|---|---|---|
| **I feel tense or 'wound up'** | most of the time | a lot of the time | only occasionally | never |
| **I still enjoy the things I used to** | just as much as ever | not quite as much | only a little | hardly at all |
| **I get a sort of frightened feeling as if something awful is about to happen** | a lot, and quite badly | sometimes, but not too badly | a little, but it doesn't worry me | never |
| **I can laugh and see the funny side of things** | as much as I always could | not quite as much as I used to | a lot less than I used to | never |
| **Worrying thoughts go through my mind** | a great deal of the time | a lot of the time | from time to time, but not often | only occasionally |
| **I feel cheerful** | never | not often | sometimes | most of the time |
| **I can sit at ease and feel relaxed** | nearly all the time | usually | not often | never |
| **I feel as if I am slowed down** | nearly all the time | very often | sometimes | never |
| **I get a sort of frightened feeling like 'butterflies' in the stomach** | never | occasionally | quite often | very often |
| **I have lost interest in my appearance** | completely | I don't care nearly as much as I should | I don't take quite as much care as I used to | I take as much care as ever |
| **I feel restless as if I have to be on the move** | very much indeed | quite a lot | not very much | never |
| **I look forward with enjoyment to things** | as much as I ever did | less than I used to | a lot less than I used to | never |
| **I get sudden feelings of panic** | very often | quite often | only occasionally | never |
| **I can enjoy a book or TV program** | often | sometimes | not often | hardly at all |

# Your feelings about yourself

**Q12. Your feelings about yourself are an important part of your health and wellbeing. Please answer the questions as in the example below.**

For example

| | *strongly agree* | *agree* | *disagree* | *strongly disagree* |
|---|---|---|---|---|
| *I am a healthy person* | ☐ | ☐ | ☑ | ☐ |

In the example someone has ticked the third box saying that they disagree that they are a healthy person.

*For EACH of the following statements please indicate how much you agree or disagree with them by ticking the box that applies.*

| | strongly agree | agree | disagree | strongly disagree |
|---|---|---|---|---|
| When I make up my mind to do something I expect to be successful | ☐ 1 | ☐ 2 | ☐ 3 | ☐ 4 |
| On the whole I am satisfied about myself | ☐ 1 | ☐ 2 | ☐ 3 | ☐ 4 |
| I wish I could have more respect for myself | ☐ 1 | ☐ 2 | ☐ 3 | ☐ 4 |
| I feel I am a person of worth, at least equal to others | ☐ 1 | ☐ 2 | ☐ 3 | ☐ 4 |
| I take a positive attitude towards myself | ☐ 1 | ☐ 2 | ☐ 3 | ☐ 4 |
| Nowadays there seem to be a lot of problems that I can't solve however hard I try | ☐ 1 | ☐ 2 | ☐ 3 | ☐ 4 |
| I am able to do things as well as most people | ☐ 1 | ☐ 2 | ☐ 3 | ☐ 4 |
| I often feel I have little control over the things that happen to me | ☐ 1 | ☐ 2 | ☐ 3 | ☐ 4 |
| All in all I am inclined to think I am a failure | ☐ 1 | ☐ 2 | ☐ 3 | ☐ 4 |
| At times I think I am no good at all | ☐ 1 | ☐ 2 | ☐ 3 | ☐ 4 |
| I feel I have a number of good qualities | ☐ 1 | ☐ 2 | ☐ 3 | ☐ 4 |
| I certainly feel useless at times | ☐ 1 | ☐ 2 | ☐ 3 | ☐ 4 |
| I feel I do not have much to be proud of | ☐ 1 | ☐ 2 | ☐ 3 | ☐ 4 |
| I can achieve all my goals if I put my mind to it | ☐ 1 | ☐ 2 | ☐ 3 | ☐ 4 |

# Your home

**Q13. Please tick the box under the face which shows how best you feel about your house or flat.**

*Please tick ONE box.*

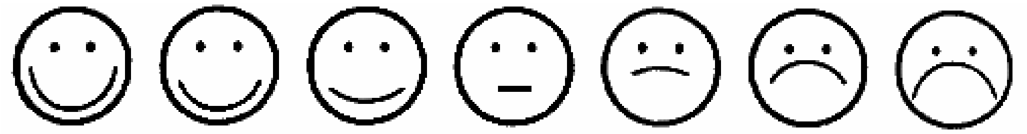

| | | | | | | |
|---|---|---|---|---|---|---|
| ☐ 1 | ☐ 2 | ☐ 3 | ☐ 4 | ☐ 5 | ☐ 6 | ☐ 7 |

**Q14. Below are some opinions that people might have about their home. How strongly do you agree or disagree with each one?**

*Please tick ONE box for EACH statement.*

| | strongly agree | agree | neither agree nor disagree | disagree | disagree strongly |
|---|---|---|---|---|---|
| I feel I have privacy in my home | ☐ 1 | ☐ 2 | ☐ 3 | ☐ 4 | ☐ 5 |
| I can get away from it all in my home | ☐ 1 | ☐ 2 | ☐ 3 | ☐ 4 | ☐ 5 |
| I can do what I want, when I want with my home | ☐ 1 | ☐ 2 | ☐ 3 | ☐ 4 | ☐ 5 |
| Most people would like a home like mine | ☐ 1 | ☐ 2 | ☐ 3 | ☐ 4 | ☐ 5 |
| I feel in control of my home | ☐ 1 | ☐ 2 | ☐ 3 | ☐ 4 | ☐ 5 |
| I feel safe in my home | ☐ 1 | ☐ 2 | ☐ 3 | ☐ 4 | ☐ 5 |
| My home makes me feel I'm doing well in life | ☐ 1 | ☐ 2 | ☐ 3 | ☐ 4 | ☐ 5 |
| I worry about losing my home | ☐ 1 | ☐ 2 | ☐ 3 | ☐ 4 | ☐ 5 |
| My home life has a sense of routine | ☐ 1 | ☐ 2 | ☐ 3 | ☐ 4 | ☐ 5 |
| My home expresses my personality and values | ☐ 1 | ☐ 2 | ☐ 3 | ☐ 4 | ☐ 5 |

# Your Household

We would like to find out about your **household**. A household is either one person living alone **OR** a group of people (not necessarily related) living at the same address with common housekeeping – sharing either a living room or sitting room, or at least one meal a day. We are interested in this because different households have different needs for transport and housing.

**Q15. Do you live alone?**

| *Please tick ONE box.* | yes ☐ 1 | no ☐ 2 |
|---|---|---|

☞ If **YES** you live alone please go to **Q17** on page 7

☞ If **NO** you stay with other people please go to **Q16** below

**Q16. Please tell us about everybody else in your household (that is anyone who has your home as their main or only home and either shares one meal a day with you or shares the living accommodation with you).**

This information is completely confidential.

*a) In the first column WRITE their relationship to you (e.g. sister or lodger). We do NOT need to know their name,*

*b) in the second column TICK the box that indicates whether they are male or female,*

*c) in the third WRITE their age in the box and*

*d) TICK the fourth column if they have a long-standing illness, disability or infirmity.*

| a) relationship to you | b) male | female | c) age | | d) do they have a long-standing illness, disability or infirmity? |
|---|---|---|---|---|---|
| Person 1 | ☐ 1 | ☐ 2 | | | ☐ 3 |
| Person 2 | ☐ 1 | ☐ 2 | | | ☐ 3 |
| Person 3 | ☐ 1 | ☐ 2 | | | ☐ 3 |
| Person 4 | ☐ 1 | ☐ 2 | | | ☐ 3 |
| Person 5 | ☐ 1 | ☐ 2 | | | ☐ 3 |
| Person 6 | ☐ 1 | ☐ 2 | | | ☐ 3 |
| Person 7 | ☐ 1 | ☐ 2 | | | ☐ 3 |
| Person 8 | ☐ 1 | ☐ 2 | | | ☐ 3 |
| Person 9 | ☐ 1 | ☐ 2 | | | ☐ 3 |
| Person 10 | ☐ 1 | ☐ 2 | | | ☐ 3 |

**Q17. Would you describe your home as a…?**

*Please tick ONE box.*

| | | | |
|---|---|---|---|
| detached house | ☐ 01 | flat in a traditional **sandstone** tenement | ☐ 06 |
| semi detached house | ☐ 02 | flat in a **modern** tenement (not sandstone) | ☐ 07 |
| terraced house | ☐ 03 | flat in a low rise block (4 floors or less) | ☐ 08 |
| flat 'four in a block' | ☐ 04 | flat in a high rise block (5 or more floors) | ☐ 09 |
| flat in a conversion | ☐ 05 | something else *(please tick box and describe below)* | ☐ 10 |

**Q18. On what floor of your building is your main living accommodation?**

*Please tick ONE box.*

| | | | |
|---|---|---|---|
| ground floor / street level | ☐ 1 | above ground floor | ☐ 3 |
| basement or semi basement | ☐ 2 | *If above ground floor please write floor level in here e.g. 5th* | ☐☐ |

**Q19. Does your household own or rent the accommodation?**

We would like to know about your household, so if you stay in a friend's home or your parents' home, for example, please tick whether THEY own or rent the accommodation.

*Please tick ONE box that applies to your household.*

| | | | |
|---|---|---|---|
| rented from the Council | ☐ 1 | being bought with a mortgage | ☐ 5 |
| rented from Glasgow Housing Association (GHA) | ☐ 2 | owned outright | ☐ 6 |
| rented from a housing association, cooperative or charitable trust | ☐ 3 | partly bought and partly rented (i.e. shared ownership) | ☐ 7 |
| rented from a private landlord or letting agency | ☐ 4 | something else *(please tick box and describe below)* | ☐ 8 |

☞ If your home is **RENTED**, please go to **Q21 on page 8**

☞ If your home is **OWNED** (or being bought), please go to **Q20** below

**Q20. Is this home an ex-council or housing association property?**

| | | | |
|---|---|---|---|
| *Please tick ONE box.* | yes ☐ 1 | no ☐ 2 | don't know ☐ 3 |

**Q21. Please count the number of rooms your household has for its own use.**

*Do not count:*
Bathrooms, toilets, halls or landings, or rooms that can only be used for storage such as cupboards.

*Do count:*
All other rooms, for example kitchens, bedrooms, living rooms, utility rooms and studies.
*If two rooms have been converted into one, count them as one room.*

*Please WRITE the number in the boxes below.*

The total number of rooms is ☐☐

**Q22. How many years have you lived in your current home?**

*Please WRITE in the boxes below.*

☐☐

**Q23. How many hours do you usually spend at home on a typical day (including time spent asleep)?**

We would like to know about a typical weekday (Monday to Friday) and a typical day at the weekend (Saturday or Sunday).

*Please WRITE the number of hours in the boxes.*

| | | |
|---|---|---|
| typical weekday | ☐☐ | hours per day (out of 24 hours) |
| typical weekend day | ☐☐ | hours per day (out of 24 hours) |

**Q24. Compared with other houses and flats in your street is your home...?**

*Please tick ONE box.*

- worth more ☐ 1
- worth about the same amount ☐ 2
- worth less ☐ 3

**Q25. Compared with other houses and flats in your street is your home...?**

*Please tick ONE box.*

- in better condition ☐ 1
- about the same ☐ 2
- worse condition ☐ 3

**Q26. Do you have a garden or yard?**

*Please tick ONE box.*

- no ☐ 1
- yes, communal or shared with at least one other household ☐ 2
- yes, not shared with any other household ☐ 3

**Q27. Do you have a dog in your household?**

*Please tick ONE box.*

- yes ☐ 1
- no ☐ 2

**Q28.** **The next question is about problems that people can have with their homes. To what extent, in your opinion, is each of the following a problem in your home?**

*Please tick ONE box for EACH problem.*

| | a serious problem | a minor problem | not a problem |
|---|---|---|---|
| damp or condensation | ☐ 1 | ☐ 2 | ☐ 3 |
| keeping your home warm in winter | ☐ 1 | ☐ 2 | ☐ 3 |
| too little space (feeling crowded) | ☐ 1 | ☐ 2 | ☐ 3 |
| too much space (too large) | ☐ 1 | ☐ 2 | ☐ 3 |
| noise from other household members | ☐ 1 | ☐ 2 | ☐ 3 |
| noise from your neighbours | ☐ 1 | ☐ 2 | ☐ 3 |
| noise from the street | ☐ 1 | ☐ 2 | ☐ 3 |
| poor state of repair | ☐ 1 | ☐ 2 | ☐ 3 |

**Q29.** **Is it ever difficult for your household to meet the cost of...?**

*Please tick ONE box on EACH line.*

| | very often | quite often | only occasionally | never | not applicable |
|---|---|---|---|---|---|
| rent or mortgage | ☐ 1 | ☐ 2 | ☐ 3 | ☐ 4 | ☐ 5 |
| repairs, maintenance and factor charges for your home | ☐ 1 | ☐ 2 | ☐ 3 | ☐ 4 | ☐ 5 |
| gas, electricity and other fuel bills | ☐ 1 | ☐ 2 | ☐ 3 | ☐ 4 | ☐ 5 |
| telephone bill | ☐ 1 | ☐ 2 | ☐ 3 | ☐ 4 | ☐ 5 |
| bills for council tax, insurance etc. that come up from time to time | ☐ 1 | ☐ 2 | ☐ 3 | ☐ 4 | ☐ 5 |
| food | ☐ 1 | ☐ 2 | ☐ 3 | ☐ 4 | ☐ 5 |

**Q30.** **We are interested in your views about home ownership, even if you rent your home. What do you think are the three BEST things about owning a home?**

Please answer this question even if you rent your home.

1. ______________________________

2. ______________________________

3. ______________________________

**Q31. What do you think are the three WORST things about owning a home?**

Please answer this question even if you rent your home.

1. ______________________________
2. ______________________________
3. ______________________________

**Q32. We are interested in your views about renting a home, even if you own your home. What do you think are the three BEST things about renting a home?**

Please answer this question even if you own your home.

1. ______________________________
2. ______________________________
3. ______________________________

**Q33. What do you think are the three WORST things about renting a home?**

Please answer this question even if you own your home.

1. ______________________________
2. ______________________________
3. ______________________________

# Your Neighbourhood

**Q34. Please TICK the box under the face which shows best how you feel about living in your neighbourhood?**

*Please tick ONE box.*

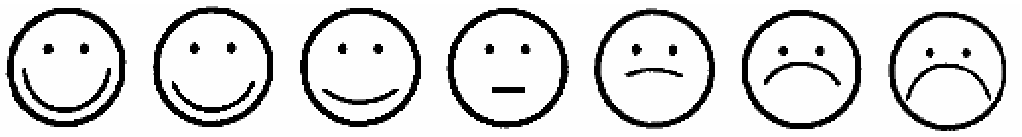

☐ 1 ☐ 2 ☐ 3 ☐ 4 ☐ 5 ☐ 6 ☐ 7

**Q35. Do you feel part of your local community?**

*Please tick ONE box.*

very much ☐ 1 a little ☐ 2 not at all ☐ 3

**Q36. How well placed do you think your home is for...?** *Please tick ONE box for EACH statement.*

| | very well placed | fairly well placed | not very well placed | not at all well placed |
|---|---|---|---|---|
| getting to work | ☐ 1 | ☐ 2 | ☐ 3 | ☐ 4 |
| general food stores | ☐ 1 | ☐ 2 | ☐ 3 | ☐ 4 |
| your doctor's surgery | ☐ 1 | ☐ 2 | ☐ 3 | ☐ 4 |
| the nearest hospital with a casualty department | ☐ 1 | ☐ 2 | ☐ 3 | ☐ 4 |
| primary schools | ☐ 1 | ☐ 2 | ☐ 3 | ☐ 4 |
| secondary schools | ☐ 1 | ☐ 2 | ☐ 3 | ☐ 4 |
| safe play areas | ☐ 1 | ☐ 2 | ☐ 3 | ☐ 4 |
| public transport/ buses and trains | ☐ 1 | ☐ 2 | ☐ 3 | ☐ 4 |
| libraries (including mobile libraries) | ☐ 1 | ☐ 2 | ☐ 3 | ☐ 4 |
| chemist or pharmacy | ☐ 1 | ☐ 2 | ☐ 3 | ☐ 4 |
| somewhere green and pleasant to walk or sit *(apart from your own garden)* | ☐ 1 | ☐ 2 | ☐ 3 | ☐ 4 |
| public recreation or sports facilities *(e.g. swimming pool, sports centre)* | ☐ 1 | ☐ 2 | ☐ 3 | ☐ 4 |

**Q37. Around where you live would you say that any of the following are a serious problem, a minor problem or not a problem?** *Please tick ONE box for EACH problem*

| | a serious problem | a minor problem | not a problem |
|---|---|---|---|
| vandalism | ☐ 1 | ☐ 2 | ☐ 3 |
| litter and rubbish | ☐ 1 | ☐ 2 | ☐ 3 |
| smells and fumes | ☐ 1 | ☐ 2 | ☐ 3 |
| assaults or muggings | ☐ 1 | ☐ 2 | ☐ 3 |
| burglaries | ☐ 1 | ☐ 2 | ☐ 3 |
| disturbance by children or youngsters | ☐ 1 | ☐ 2 | ☐ 3 |
| speeding traffic | ☐ 1 | ☐ 2 | ☐ 3 |
| discarded needles or syringes | ☐ 1 | ☐ 2 | ☐ 3 |
| uneven or dangerous pavements | ☐ 1 | ☐ 2 | ☐ 3 |
| nuisance from dogs | ☐ 1 | ☐ 2 | ☐ 3 |
| reputation of neighbourhood | ☐ 1 | ☐ 2 | ☐ 3 |
| poor public transport | ☐ 1 | ☐ 2 | ☐ 3 |
| noise | ☐ 1 | ☐ 2 | ☐ 3 |
| the people round here | ☐ 1 | ☐ 2 | ☐ 3 |

**Q38. How many people are there in your neighbourhood with whom you exchange small favours?**

An example would be leaving a key to let a repair man in.

*Please WRITE the number of people in the boxes.*

I exchange favours with ☐☐ people who live in my neighbourhood.

**Q39. How many of your neighbours do you know by name?**

*Please WRITE the number of people in the boxes.*

☐☐

## Your transport

**Q40. Please TICK the box under the face which shows best how you feel about the means of transport that you normally use to get around.**

*Please tick ONE box.*

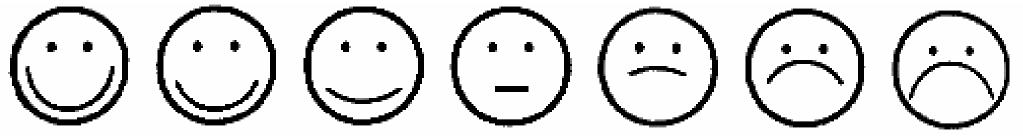

| | | | | | | |
|---|---|---|---|---|---|---|
| ☐ 1 | ☐ 2 | ☐ 3 | ☐ 4 | ☐ 5 | ☐ 6 | ☐ 7 |

**Q41. How many cars or vans are owned, or available for use, by members of your household?**

Include company cars/vans if private use allowed and exclude vans used solely for carrying goods.

*Please tick ONE box.*

| | | | | |
|---|---|---|---|---|
| none ☐ 0 | one ☐ 1 | two ☐ 2 | three ☐ 3 | four or more ☐ 4 |

If four or more *please WRITE number in here* ☐☐

☞ If there are **NONE** please go to **Q44** on page 13

☞ If there are **ONE OR MORE** please go to **Q42** below

**Q42. Can we just check, are ALL of these cars and vans owned by or leased to people who live in your household, rather than owned by or leased to someone living somewhere else?**

*Please tick ONE box.* yes ☐ 1 no ☐ 2

**Q43. Please tell us about the cars and vans that are owned or leased to your household.**

Please start with the car or van you use most. So if you have one car, please just fill in details for car or van 1.

*Please WRITE in the make and the model, the year of manufacture and also the amount you think it is worth as in the example shown.*

| | a) make | b) model | c) year of manufacture | d) amount worth |
|---|---|---|---|---|
| *Example* | Ford | Fiesta | 2002 | £ 1800 |
| Car or van 1 | | | | £ |
| Car or van 2 | | | | £ |
| Car or van 3 | | | | £ |
| Car or van 4 | | | | £ |

***From the list of cars above, which is the household's MAIN car or van?***

**e) Which car or van is it…?**

*Please tick ONE box* car or van 1 ☐1 car or van 2 ☐2 car or van 3 ☐3 car or van 4 ☐4

**f) Was this car or van acquired…?**

*Please tick ONE box* new ☐1 second-hand ☐2 as a company car ☐3

**g) Compared with other cars or vans in your neighbourhood is this car or van worth more, about the same or less?**

*Please tick ONE box* worth more ☐1 worth about the same ☐2 worth less ☐3

**Some** of the next questions talk about **public transport**. By public transport we mean buses, coaches, trains and underground trains.

**Q44. How do you usually travel to the following?** *Please tick ALL that you usually use for EACH destination.*

| | I don't go | car or van | public transport | taxi | walk | cycle |
|---|---|---|---|---|---|---|
| health appointments | ☐1 | ☐2 | ☐3 | ☐4 | ☐5 | ☐6 |
| supermarket | ☐1 | ☐2 | ☐3 | ☐4 | ☐5 | ☐6 |
| sports facilities | ☐1 | ☐2 | ☐3 | ☐4 | ☐5 | ☐6 |
| family/friends | ☐1 | ☐2 | ☐3 | ☐4 | ☐5 | ☐6 |
| days out | ☐1 | ☐2 | ☐3 | ☐4 | ☐5 | ☐6 |
| evenings out | ☐1 | ☐2 | ☐3 | ☐4 | ☐5 | ☐6 |
| work/college | ☐1 | ☐2 | ☐3 | ☐4 | ☐5 | ☐6 |
| taking children to school | ☐1 | ☐2 | ☐3 | ☐4 | ☐5 | ☐6 |

**Q45. How easy is it for you to travel to the following using your usual form of transport?**

*Please tick ONE box for EACH destination.*

| | I don't go | very easy | quite easy | quite difficult | very difficult |
|---|---|---|---|---|---|
| health appointments | ☐ 1 | ☐ 2 | ☐ 3 | ☐ 4 | ☐ 5 |
| supermarket | ☐ 1 | ☐ 2 | ☐ 3 | ☐ 4 | ☐ 5 |
| sports facilities | ☐ 1 | ☐ 2 | ☐ 3 | ☐ 4 | ☐ 5 |
| family/friends | ☐ 1 | ☐ 2 | ☐ 3 | ☐ 4 | ☐ 5 |
| days out | ☐ 1 | ☐ 2 | ☐ 3 | ☐ 4 | ☐ 5 |
| evenings out | ☐ 1 | ☐ 2 | ☐ 3 | ☐ 4 | ☐ 5 |
| work/college | ☐ 1 | ☐ 2 | ☐ 3 | ☐ 4 | ☐ 5 |
| taking children to school | ☐ 1 | ☐ 2 | ☐ 3 | ☐ 4 | ☐ 5 |

**Q46. How often is there a car or van available when you need to drive it or have a lift?**

*Please tick ONE box.*

| always | most of the time | some of the time | occasionally | never |
|---|---|---|---|---|
| ☐ 1 | ☐ 2 | ☐ 3 | ☐ 4 | ☐ 5 |

☞ If you **NEVER** travel by car or van please go to **Q50** on page 15

☞ If you **EVER** travel by cars and vans please go to **Q47** below

**Q47. How long would you spend in a car or van on a typical day?**

Please don't include time spent as part of your paid work.

If on a typical day you spend no time please write 0.

*Please WRITE in the boxes the number of hours and minutes you would spend out of 24 hours*

.

| | | | | |
|---|---|---|---|---|
| typical weekday | ☐☐ | hours | ☐☐ | mins |
| typical weekend day | ☐☐ | hours | ☐☐ | mins |

**Q48. When you travel by car are you USUALLY...?**

*Please tick ONE box.*

| a driver | a passenger | sometimes a driver, sometimes a passenger |
|---|---|---|
| ☐ 1 | ☐ 2 | ☐ 3 |

*BARCODE 1* *CONFIDENTIAL*

**Q49. The next question looks at feelings people might have about travelling by car or van. How much do you agree or disagree with each statement?**

Please answer all the questions if you ever, even if only occasionally, travel by car or van.

*Please tick ONE box for EACH statement.*

| | strongly agree | agree | neither agree nor disagree | disagree | disagree strongly |
|---|---|---|---|---|---|
| I feel I have privacy when I'm in a car or van | ☐ 1 | ☐ 2 | ☐ 3 | ☐ 4 | ☐ 5 |
| I feel I can get away from stresses as I travel by car or van | ☐ 1 | ☐ 2 | ☐ 3 | ☐ 4 | ☐ 5 |
| I can travel where I want, when I want by car or van | ☐ 1 | ☐ 2 | ☐ 3 | ☐ 4 | ☐ 5 |
| Most people would like a car or van like the one that I usually use | ☐ 1 | ☐ 2 | ☐ 3 | ☐ 4 | ☐ 5 |
| I feel in control when I travel by car or van | ☐ 1 | ☐ 2 | ☐ 3 | ☐ 4 | ☐ 5 |
| I feel safe when I travel by car or van | ☐ 1 | ☐ 2 | ☐ 3 | ☐ 4 | ☐ 5 |
| When I travel by car or van it makes me feel I'm doing well in life | ☐ 1 | ☐ 2 | ☐ 3 | ☐ 4 | ☐ 5 |
| I worry about the car or van I use having to be sold | ☐ 1 | ☐ 2 | ☐ 3 | ☐ 4 | ☐ 5 |
| Travelling by car or van fits in well with the routine of my daily life | ☐ 1 | ☐ 2 | ☐ 3 | ☐ 4 | ☐ 5 |
| Travelling by car or van expresses my personality and values | ☐ 1 | ☐ 2 | ☐ 3 | ☐ 4 | ☐ 5 |

**Q50. Do you EVER travel by public transport?**

That is buses, coaches, trains and underground trains.

*Please tick ONE box.*

| | | | | |
|---|---|---|---|---|
| very often ☐ 1 | quite often ☐ 2 | sometimes ☐ 3 | occasionally ☐ 4 | never ☐ 5 |

☞ If you **NEVER** travel by public transport please go to **Q53** on page 16

☞ If you **EVER** travel by public transport please go to **Q51** below

**Q51. How long would you spend on public transport on a typical day?**

Please don't include time spent as part of your paid work.

If on a typical day you spend no time please write 0.

*Please WRITE the number of hours and minutes you would spend out of 24 hours in the boxes.*

| | | | | |
|---|---|---|---|---|
| typical weekday | ☐☐ | hours | ☐☐ | mins |
| typical weekend day | ☐☐ | hours | ☐☐ | mins |

**Q52. This question is about general feelings about public transport. How much do you agree or disagree with each statement?**

Please answer all the questions if you ever, even if only occasionally, travel by public transport.

*Please tick ONE box for EACH statement.*

| | strongly agree | agree | neither agree nor disagree | disagree | disagree strongly |
|---|---|---|---|---|---|
| I feel I have privacy when I travel by public transport | ☐ 1 | ☐ 2 | ☐ 3 | ☐ 4 | ☐ 5 |
| I feel I can get away from stresses when I travel by public transport | ☐ 1 | ☐ 2 | ☐ 3 | ☐ 4 | ☐ 5 |
| I can travel where I want, when I want by public transport | ☐ 1 | ☐ 2 | ☐ 3 | ☐ 4 | ☐ 5 |
| Most people would like to travel by the public transport that I use | ☐ 1 | ☐ 2 | ☐ 3 | ☐ 4 | ☐ 5 |
| I feel in control when I use public transport | ☐ 1 | ☐ 2 | ☐ 3 | ☐ 4 | ☐ 5 |
| I feel safe when I travel by public transport | ☐ 1 | ☐ 2 | ☐ 3 | ☐ 4 | ☐ 5 |
| When I travel by public transport it makes me feel that I'm doing well in life | ☐ 1 | ☐ 2 | ☐ 3 | ☐ 4 | ☐ 5 |
| I worry about bus/train services being changed or dropped | ☐ 1 | ☐ 2 | ☐ 3 | ☐ 4 | ☐ 5 |
| Public transport times fit in well with the routine of my daily life | ☐ 1 | ☐ 2 | ☐ 3 | ☐ 4 | ☐ 5 |
| Public transport expresses my personality and values | ☐ 1 | ☐ 2 | ☐ 3 | ☐ 4 | ☐ 5 |

**Q53. What do you think are the three BEST things about public transport?**

Please answer this question even if you never travel by public transport.

1. ____________________
2. ____________________
3. ____________________

**Q54. What do you think are the three WORST things about public transport?**

Please answer this question even if you never travel by public transport.

1. ____________________
2. ____________________
3. ____________________

**Q55. What do you think are the three BEST things about having a car?**

Please answer this question even if you never travel by car.

1. ______________________________
2. ______________________________
3. ______________________________

**Q56. What do you think are the three WORST things about having a car?**

Please answer this question even if you never travel by car.

1. ______________________________
2. ______________________________
3. ______________________________

# Work

Whether people work is often an important aspect of people's lives and may affect their transport and housing, so we would like to ask you about your situation.

**Q57. Which of these comes closest to how you would describe yourself at present?**

*Please tick ONE box.*

| | | | |
|---|---|---|---|
| doing paid work full time | ☐ 1 | disabled, invalid or permanently sick | ☐ 6 |
| doing paid work part time | ☐ 2 | caring for home and family or dependants | ☐ 7 |
| on a government training scheme | ☐ 3 | full time student | ☐ 8 |
| retired | ☐ 4 | something else *(please tick and describe below)* | ☐ 9 |
| unemployed | ☐ 5 | | |

**Q58. If you are NOT currently in paid work have you EVER been in paid work?**

| | | |
|---|---|---|
| *Please tick ONE box.* | yes ☐ 1 | no ☐ 2 |

☞ If you have **never done paid work** please go to **Q66** on page 19

☞ If you have **ever done paid work** please go to **Q59** on page 18

**Q59. Please WRITE the title of your present paid job (or if you are not currently working your most recent job), describe what you actually do (did) and what sort of employer you work or worked for**

Job title (e.g. assistant chef)

Job description (e.g. make puddings, supervise dish washing)

Type of employer (e.g. school)

**Q60. Which of these best describes your current work (or most recent work if not currently working)?**

*Please tick ONE box.*

| | | | | | |
|---|---|---|---|---|---|
| self employed with paid employees | ☐ 1 | manager | ☐ 3 | employee | ☐ 5 |
| self employed with no paid employees | ☐ 2 | foreman/supervisor | ☐ 4 | | |

**Q61. What size of organisation do or did you work in?**

*Please tick ONE box.*

| | | | |
|---|---|---|---|
| a large organisation (25 or more employees) | ☐ 1 | a small organisation (fewer than 25 employees) | ☐ 2 |

**Q62. How far away is or was your work from your home?**

*Please WRITE the number of miles in the boxes below.*

☐☐ mile(s)

**Q63. How long does or did it take you to get to work?**

*Please WRITE the number of hours and minutes in the boxes below.*

☐☐ hours ☐☐ mins

**Q64. We are interested to know whether people who work in different places have different problems getting to work so we would like to know the post code of your workplace.**

If you do not know the whole postcode please just write in the parts that you do know.

*Please WRITE the postcode in the boxes below as in the example postcode, ML1 2AB.*

| *e.g.* | | M | L | 1 | - | 2 | A | B |
|---|---|---|---|---|---|---|---|---|
| | | | | | - | | | |

**Q65. How much time do or did you spend travelling as part of your job on a typical day?**

*Please WRITE the number of hours and minutes out of 24 hours in the boxes below.*

☐☐ hours ☐☐ mins

**Q66. Do you have a spouse or partner who has ever been in paid work?**

*Please tick ONE box.*

| | | |
|---|---|---|
| yes ☐ 1 | no ☐ 2 | not applicable ☐ 3 |

☞ If **NO (or not applicable)** please go to **Q71** on page 20

☞ If **YES** please go to **Q67 below**

**Q67. Which of these comes closest to how you would describe your spouse or partner's situation at present (if applicable)?** *Please tick ONE box.*

| | | | |
|---|---|---|---|
| doing paid work full time | ☐ 1 | unemployed | ☐ 6 |
| doing paid work part time | ☐ 2 | disabled, invalid or permanently sick | ☐ 7 |
| on a government training scheme | ☐ 3 | caring for home and family or dependants | ☐ 8 |
| retired | ☐ 4 | something else *(please tick and describe below)* | ☐ 9 |
| full time student | ☐ 5 | | |

**Q68. Please WRITE the title of your spouse or partner's present paid work (or most recent paid job if they are not currently working) describe what they actually do (did) and the type of employer they work or used to work for.**

Job title (e.g. cleaner)

Job description (e.g. clean factory)

Type of employer (e.g. chemical manufacturer)

**Q69. Which of these best describes the current work or most recent work of your spouse or partner?**

*Please tick ONE box.*

| | | | | | |
|---|---|---|---|---|---|
| self employed with paid employees | ☐ 1 | manager | ☐ 3 | employee | ☐ 5 |
| self employed with no paid employees | ☐ 2 | foreman/supervisor | ☐ 4 | | |

***BARCODE 1*** ***CONFIDENTIAL***

**Q70.** **What size of organisation does or did your spouse or partner work for?**

| | | | | |
|---|---|---|---|---|
| *Please tick ONE box.* | a large organisation (25 or more employees) | ☐ 1 | a small organisation (fewer than 25 employees) | ☐ 2 |

## Money matters

**Q71.** **How much are the mortgage or rent payments for your home per month?**

Please don't include Council Tax payments.

Please do include amounts paid by the government as benefits.

*Please WRITE the amount in the boxes.*

£ ☐☐☐☐☐ per month

**Q72.** **What is the total NET income of everyone in your household (including yourself) altogether per month?**

Please include benefits.

Please tell us about take-home pay (after tax and National Insurance).

*Please WRITE the amount in the boxes.*

£ ☐☐☐☐☐ per month

**Q73.** **What proportion of your household income (including your own) would you say comes from benefits?**

*Please tick ONE box.*

| | | | | | |
|---|---|---|---|---|---|
| none ☐ 1 | very little ☐ 2 | about a quarter ☐ 3 | about half ☐ 4 | about three quarters ☐ 5 | all ☐ 6 |

## Lifestyles

In this final section we would like to find out about aspects of people's lifestyles which may affect their health.

**Q74.** **Do you smoke now, even if it is just occasionally, or have you ever smoked in the past?**

| | | | |
|---|---|---|---|
| *Please tick ONE box.* | smoke now ☐ 1 | in past only ☐ 2 | never ☐ 3 |

**Q75.** **What about exercise? On how many days in an average month (4 weeks) do you do any sport or physical exercise (e.g. dancing or brisk walking) that makes you out of breath and sweat, and that you do for more than 20 minutes at a time?**

*Please WRITE the number of days a month in the boxes.*

☐☐ days in an average month

**Q76. During the last year, have you done any walks of 2 miles or more? These are walks which would usually take about 40 minutes.** *We are interested both in walks you took for pleasure and in walking for other reasons, like to and from work, or to the shops.*

*Please tick ONE box.* yes ☐ 1 no ☐ 2

☞ If **NO** please go to **Q78** below

☞ If **YES** please go to **Q77** below

**Q77. If yes, is that mainly around your neighbourhood that is *in* the area, *outside* the area, or *both*?**

*Please tick ONE box.* in the area ☐ 1 outside the area ☐ 2 both ☐ 3

**Q78. Is there anything else that you would like to tell us?**

If you have any other comments that you would like to make, please write it in the box below.

______________________________

______________________________

______________________________

______________________________

**THANK YOU VERY MUCH FOR COMPLETING THIS QUESTIONNAIRE. We could not do this study without your help.**

***Please could you just look back to check that you haven't missed any questions by mistake or turned two pages at once.***

**Now please send it back to us in the envelope provided.**
